# Neck Circumference to Assess Obesity in Preschool Children

**DOI:** 10.4274/jcrpe.3525

**Published:** 2017-03-01

**Authors:** Meda Kondolot, Duygu Horoz, Serpil Poyrazoğlu, Arda Borlu, Ahmet Öztürk, Selim Kurtoğlu, Mümtaz M. Mazıcıoğlu

**Affiliations:** 1 Erciyes University Faculty of Medicine, Department of Pediatrics, Social Pediatrics Unit, Kayseri, Turkey; 2 The Head of Local Health Authority, Kayseri, Turkey; 3 Erciyes University Faculty of Medicine, Department of Public Health, Kayseri, Turkey; 4 Erciyes University Faculty of Medicine, Department of Biostatistics, Kayseri, Turkey; 5 Erciyes University Faculty of Medicine, Department of Pediatric Endocrinology, Kayseri, Turkey; 6 Erciyes University Faculty of Medicine, Department of Family Medicine, Kayseri, Turkey

**Keywords:** Preschool children, obesity, neck circumference, percentiles, cut-offs

## Abstract

**Objective:**

Limited information is available about the use of neck circumference (NC) to assess obesity in preschool children. This study aims to provide NC percentiles and determine the cut-off levels of NC as a measure to assess obesity in preschool children.

**Methods:**

The data were obtained from the Anthropometry of Turkish Children aged 0-6 years (ATCA-06) study database. A total of 21 family health centers were chosen and children aged 2-6 years old from all socioeconomic levels were randomly selected from the lists of district midwives; 1766 children (874 male and 892 female; 88.3% of sample size) were included in the study. The smoothed centile curves of NC were constructed by the LMS method. Receiver operating characteristic (ROC) analysis was performed to calculate cut-off points for NC using body mass index ≥95th percentile.

**Results:**

Mean NC was greater in males than females. Cut-off values for obesity were found to be statistically significant in both genders other than 3 years old boys. The NC percentiles of Turkish preschool children were slightly greater than those of other European preschool children in both genders. This difference disappeared around the adiposity rebound period. The 97th percentile values for Turkish preschool children continue to be greater in both genders.

**Conclusion:**

NC may be useful to define obesity in preschool children. Since ethnic and various other factors may have a role in incidence of obesity, local reference data are important in assessment of obesity.

**WHAT IS ALREADY KNOWN ON THIS TOPIC?**

Neck circumference (NC) has been shown to be one of the reliable and practical additional measurements to define obesity in school children and adolescents. Limited information is available about the use of NC in preschool children.

**WHAT THIS STUDY ADDS?**

NC may be useful to define obesity as an additional measurement in preschool children as well.

## INTRODUCTION

Obesity in early childhood is on the increase and this is of great concern because of the relationship between childhood obesity and metabolic complications and other clinical comorbidities encountered in adult life (1). In the United States, obesity prevalence was reported to be 12.4% for boys and 10% for girls in a recent study in children aged 3 to 5 years (2). In our data set, obesity prevalence was identical in both genders as 5% in Turkish children aged 0-84 months (3). It was reported that children at the 50^th^ percentile of body-mass index (BMI) at the age of 5 years had a 6% probability of being obese at the age of 14 years and that this probability increased to 25% among 5-year-olds at the 85th percentile and to 47% among those at the 95th percentile (1). Since early childhood adiposity may lead to cardiometabolic health effects in later years, it should be closely monitored to prevent these complications (4,5,6,7,8).

Children have a rapid increase in adiposity during the first year of life. After infancy, adiposity declines and reaches a minimum and rebound at around 5 to 6 years. Subsequently, adiposity shows a steady increase throughout childhood and adolescence (6). In monitoring for obesity, it is important to follow the course of adiposity and identify the age at which excess weight gain occurs. Although BMI is a frequently used measurement to asses total body fat content, it fails to provide sufficient information regarding body fat distribution. Since upper body adiposity is considered to be a significant determinant of cardiometabolic risk factors, it should be monitored rather than assessing total body fat content. Recent studies have shown that neck circumference (NC) is one of the reliable and practical anthropometric measurements to assess upper body adiposity and consequently to assess cardiometabolic risk as a result of irregular fat distribution (9,10,11,12,13,14,15,16,17,18,19).

There are only a few studies that provide reference data on NC measurements for school children and adolescents (20,21). NC reference values for preschool children are especially limited (21).

This present study aims to 1) provide NC percentiles and determine cut-off levels of NC for obesity in preschool children; 2) to explore the importance of NC measurements as an additional tool for assessment of obesity in preschool children.

## METHODS

The data were obtained from the Anthropometry of Turkish Children aged 0-6 years (ATCA-06) study database. The data were collected between September 2009 to May 2010 in a large city in Central Turkey with a population of over 1,200,000. A two-stage probability sampling was used to select children from each socioeconomic level. In the first stage, Family Medicine Centers (FMCs) located in the city center and suburbs were selected randomly. These Centers address a population of all socioeconomic levels (low, medium, high). In determining socioeconomic levels, we used the evaluation of local health authorities. A total of 21 FMCs were chosen, and children of ages 2-6 years from these centers were randomly selected from the district midwives’ lists according to their families’ income. Parents along with their children were invited to the FMC. Children whose parents did not consent to participate were excluded from the study.

A total of 2000 children (975 male and 1025 female) were recruited for the study. Children with chronic medical disorders, cervical lymphadenomegaly, neck deformity, and those whose NC measurements were out of range for each gender and age (3^rd^-97^th^ percentiles) were excluded to obtain normally distributed data. Upon exclusion of those participants who did not match the selection criteria, a total number of 1766 children (874 male and 892 female; 88.3% of sample size) were included in the study.

Chronological age which was calculated by subtracting date of birth from date of observation was used to determine cut-offs. Each quarter year elapsed from their birthday was used to obtain percentiles in short periods. The Ethics Committee of Erciyes University approved this study (2008/28). Written parental consent was obtained prior to the study.

All measurements were performed by well-trained health technicians. NC was measured using a plastic tape measure while the child’s head was held erect with eyes facing forward and the neck in a horizontal plane at the level of the most prominent portion, the thyroid cartilage. All measurements were taken with the subjects standing upright, with the face directed forward and shoulders relaxed. BMI was calculated according to the formula: weight (kg)/height (m)^2^. Children whose BMI was ≥95^th^ percentile curve according to local references were identified as obese (3).

Construction of the percentile curves was performed with the LMS Chart Maker Pro version 2.3 software program (The Institute of Child Health, London) which fits smooth percentile curves to reference data. The smoothed percentile curves of NC were constructed by the LMS method. This method summarizes percentiles at each age based on the power of age-specific Box-Cox power transformations that are used to normalize data (22). These three quantities depend on age. The final curves of percentiles are produced by three smooth curves representing L (Lambda; skewness), M (M; median), and S (Sigma; coefficient of variation). With estimates of L, M, and S, values of X are connected to the values of z through the above equation. The percentile is obtained from a normal distribution table, where the z-score corresponds to the percentile of interest. In boys, the effective degrees of freedom (edf) for NC were equal to 3 (M curve), 4 (S curve), and 3 (L curve). In girls, edf for NC were equal to 3, 4, and 2, respectively. The median curves of boys and girls were compared to show gender-specific trends through 3-6 years.

### Statistical Analysis

Construction of the centile curves was performed with the LMS Chart Maker Pro version 2.3 software program (The Institute of Child Health, London) which fits smooth centile curves to reference data (22). Gender difference in NC was compared with student’s t-test. NC cutt-off values were calculated for 3-6-year-old children with receiver operating characteristics (ROC) analysis according to dependent variable obesity defined by BMI ≥95^th^ percentile (23). The ROC curves demonstrated the overall discriminatory power of a diagnostic test: NC. Sensitivity and specificity were calculated to identify the optimal cut-off values. Calculated cut-offs were checked with Youden index as (J) ≥0.6 good and (J) ≥0.4 moderate. Descriptive statistics for each quarter year (e.g., 3-8 m, etc.) within sex were calculated by SPSS version 15.0 (Chicago, Illinois, USA).

## RESULTS

Mean (standard deviation) and median (minimum-maximum) values for NC in Turkish children age of 2-6 years in both genders and comparisons of the means are shown in Table 1. Mean NC values were greater in males than females, and the difference was significant at age groups 30-32, 42-44, 48-50, 57-68, and 72-83 months. The mean increment from 24 to 83 months period was 1.4 cm for males and 0.5 cm for females (Table 1).

The calculated age-specific (at 3-month intervals) 3^rd^, 5^th^, 10^th^, 15^th^, 25^th^, 50^th^, 75^th^, 85^th^, 90^th^, 95^th^, and 97^th^ percntiles for NC in each gender are given in Tables 2 and 3. The increase in NC for 50^th^ percentile values through the 24 to 83 months period was 1.2 cm in boys and 0.6 in girls. The increase in NC through the 3^rd^ to 97^th^ percentiles for 24-26 months was 5.48 cm in boys. This value was 5.05 cm for 81-83 months (Table 2). The increases in NC through 3^rd^ to 97^th^ percentiles for 24-26 months and for 81-83 months in girls were 5.69 cm and 4.80 cm (Table 3).

ROC analysis was performed to calculate cut-off points for NC using BMI ≥95^th^ percentile (for obesity) as a dependent variables. The results for boys and girls are shown in Tables 4 and 5. Youden index was also calculated for cut-off values and it was found to be statistically significant in both genders except 3 years old group of boys (Tables 4, 5). In case of obesity (BMI ≥95^th^), we found that cut-off points for NC were 25.9 and 27.0 cm for males who were 4 and 5 years old, respectively (a cut-off point calculated by ROC analysis and confirmed by Youden index ≥0.4) and that these values were 27.5 cm for males who were 6 years old (Youden index ≥0.6) (Table 4). Cut-off points for NC were 25.8 (Youden index ≥0.6), 25.8 (Youden index ≥0.6), 25.7 (Youden index ≥0.4), and 25.5 cm (Youden index ≥0.4) for females who were 3, 4, 5, and 6 years old, respectively (Table 5).

**Figure 1:**
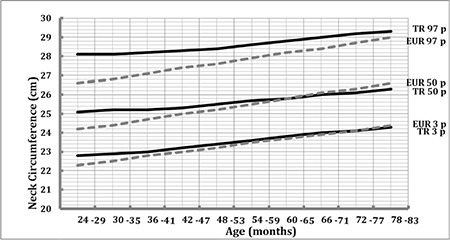
The comparison of 3^rd^, 50^th^, and 97^th^ percentiles of neck circumference in boys with European children (Identification and prevention of Dietary- and lifestyle-induced health EFfects in Children and infantS study)

**Figure 2:**
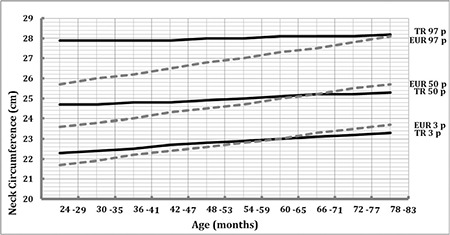
Comparison of 3^rd^, 50^th^, and 97^th^ percentiles of neck circumference in the girls with European children (Identification and prevention of Dietary- and lifestyle-induced health EFfects in Children and infantS study)

Figures 1 and 2 show comparisons of the 3^rd^, 50^th^, 97^th^ NC percentiles of our data with the IDEFICS (Identification and prevention of Dietary- and lifestyle-induced health EFfects in Children and infantS) study (conducted on European children) in boys and girls, respectively. The data indicate that NC values of Turkish preschool children were slightly greater than those of other European preschool children in both genders. This difference disappears around the adiposity rebound period, and NC values of Turkish preschool children become slightly lower after this period except for the 97^th^ percentile. This value continues to be greater in the Turkish preschool children in both genders as compared to the European data.

## DISCUSSION

BMI is obviously the most commonly used anthropometric measure to assess obesity. However, BMI may fail to describe body fat distribution. Since upper body fat deposition is associated with an increased metabolic and cardiovascular risk (24,25,26), it is absolutely essential to determine fat accumulation in the upper body. Various anthropometric measures and indices may be used to determine upper body fat content and distribution such as waist circumference (WC), mid-upper arm circumference, skinfold thickness, waist/hip ratio, and waist/height ratio. WC is the most frequently used measure, which may have some measuring limitations such as its relationship with mid expiratory movement and consumption of excess food (9,15,27,28,29). Recently, NC has been shown as a reliable and easy alternative tool to WC for determining upper body fat accumulation and distribution (11,12,13,17,18,19). The advantages of measuring NC compared with WC are better inter- and intra-observer reliability, relative stability which is less affected by respiration and clothing (11,30).

In addition, it has been reported that NC may be used to assess both obesity and metabolic disorders (12,13). NC percentiles were also produced for school children and adolescents (20,21). However, information on preschool children is limited. Cunningham et al (1) have emphasized the importance of screening predominantly in preschool children. These authors have shown that the incidence of obesity between the ages of 5 and 14 years was 4 times higher in children who had been overweight at the age of 5 years compared with those of normal weight at that age. Therefore, it is essential to assess adiposity and unstable fat distribution before and during the adiposity rebound period (4,6,7,8). Since it was shown that early childhood adiposity has certain cardiologic and metabolic health consequences in later life, we need a reliable and easy-to-use measure for preschool children.

The available data about NC percentiles of preschool children was from a multicenter study (Sweden, Germany, Hungary, Italy, Cyprus, Spain, Belgium, Estonia) that included normal-weight 2.0-10.9-year-old European children (21). Formisano et al (18) later worked on these data and showed that cardiometabolic risk was associated with increased NC. We believe that this current study will be contributory to the importance of the role of NC measurements in young children by providing local reference data as well as by comparing these references with the most recent and reliable studies in Europe.

In our study, similar to data reported by Nagy et al (21) for children in the multicenter study, we found that NC values increase with age and that they are slightly higher in males. This gender difference becomes significant in children older than 5 years. However, the increase in NC through 24 to 83 months is relatively smaller than that reported by Nagy et al (21). The increase in 50^th^ percentile NC values was 1.2 cm and 0.6 cm in our study, while the above authors reported an increase of 2.4 cm and 2.1 cm for boys and girls, respectively. Furthermore, NC percentile values in Turkish preschool children were slightly greater than those of the European preschool children in both genders (Figures 1, 2). This difference disappears around the rebound adiposity period and then NC values of Turkish preschool children decrease slightly. However, the 97^th^ percentile NC values of Turkish preschool children continue to be greater than those of their European counterparts in both genders. These findings may be related to several factors such as sample selection, ethnic or geographical differences. The study cited above (21) included data from eight European countries. However, we do not know whether similar differences would exist if our data could have been compared with findings from each individual country separately. We must also note that the similarity of NC values during the rebound adiposity period found in our study slightly differs from those cited in the above study (Figures 1, 2).

We consider that this difference may be explained by environmental and other unknown factors that could have more predominant effects than genetic factors at the adiposity rebound period and later. This should be investigated in further studies.

This study provides NC cut-off values for obesity in Turkish preschool children. Additionally, it is the first study that reports NC percentiles and cut-offs in Turkish preschool children. The power of this study may be the stratification for socioeconomic level in a relatively big sample size which may represent Turkish population. Our findings show that NC may also be a useful tool to assess obesity in preschool children in addition to BMI, since it represents upper body fat distribution. However, the cut-off values of NC established in our study should be confirmed in subsequent studies.

In conclusion, this study provides reference and cut-off values for preschool Turkish children. Since NC findings may indicate future cardiometabolic risk, both our reference and cut-off values for NC may be useful for screening and follow-up.

## Figures and Tables

**Table 1:**
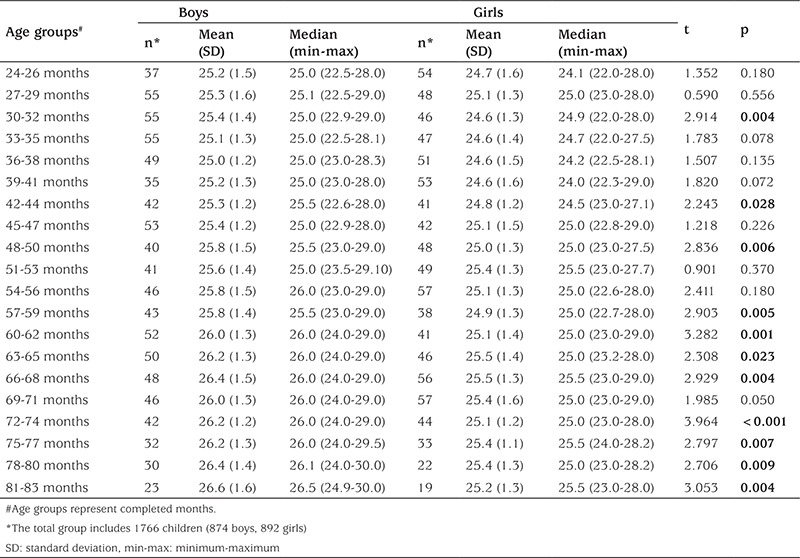
Mean (standard deviation), median (minimum-maximum) neck circumference values for Turkish children aged 2-6 years and comparisons of the means

**Table 2:**
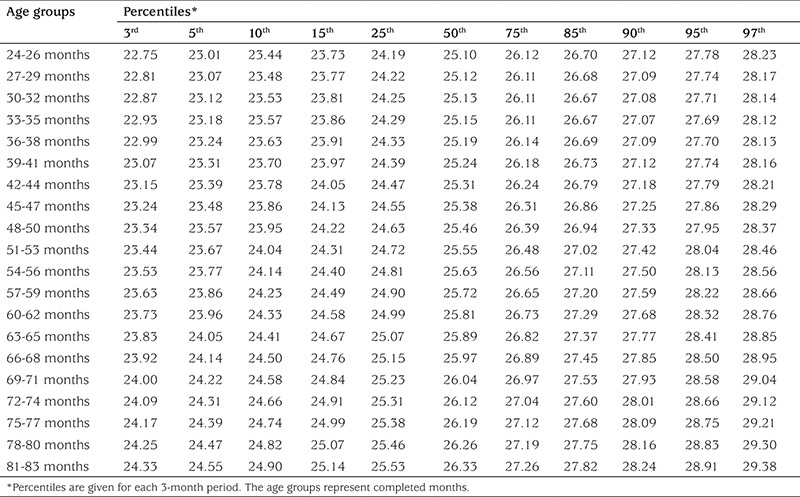
The neck circumference percentile values (3rd to 97th percentiles) for 2-6 years old boys

**Table 3:**
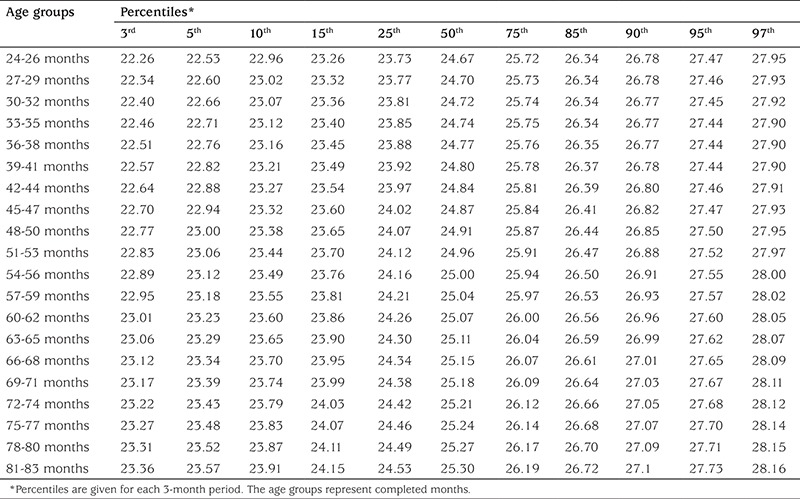
The neck circumference percentile values (3rd to 97th percentiles) for 2-6 years old girls

**Table 4:**
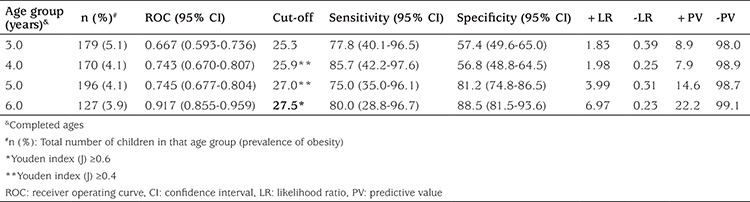
Receiver operating curve analysis for neck circumference to determine cut-off points for obesity for 3-6-year-old boys

**Table 5:**
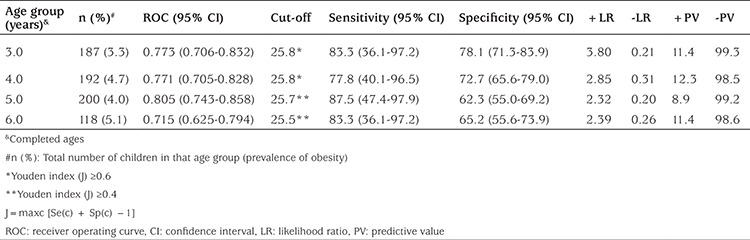
Receiver operating curve analysis for neck circumference to determine cut-off points for obesity for 3-6-year-old girls
